# Hospitals in India

**Published:** 1894-08-25

**Authors:** 


### HOSPITALS IN INDIA.

### II.—THE MADRAS PRESIDENCY.

### 2. Civil Hospitals and Dispensaries in the Madras Presidency.

In the twenty-two Revenue districts of the Madras Presidency, with an aggregate population of 34,336,196 persons, there were 414 dispensaries in operation at the end of 1891, showing a ratio of ·012 dispensaries per 1,000 of population. As compared with 1888, this shows an increase of 53 institutions, and the large increase in the number of sick treated points to a steadily increasing appreciation by the natives of European medical treatment. The following statement summarises the position of these institutions in 1888 and in 1891. It should be said that of the total number of dispensaries only 167 are provided with accommodation for in-patients, while 24 others have a certain accommodation available for cases of emergency.

| Year. | No. of Institutions. | No. of In-patients. | No. of Out-patients. | No. of beds maintained. | Daily average of indoor and outdoor patients. | Midwives. | | Surgical operations. | | Receipts. | Expenditure. | Percentage of total expenditure defrayed by Government. |
|---|---|---|---|---|---|---|---|---|---|---|---|---|
| | | | | | | No. | Cases attended. | Major. | Minor. | Rs. | Rs. | |
| 1888 | 361 | 31,496 | 2,182,723 | 2,657 | 16,193 | 157 | 6,086 | 3,075 | 64,760 | 7,07,700 | 6,99,008 | 11·76 |
| 1891 | 414 | 37,243 | 2,706,176 | 2,913 | 19,311 | 208 | 11,703 | 3,646 | 89,793 | 8,35,560 | 8,27,899 | 8·04 |

This table presents, in a complete and simple form, the most important features of a bulky report. The main difficulty in the position of these institutions appears to consist in the inadequate supply of commissioned medical officers. Young officers on joining the department are at once absorbed by the military department without any corresponding number being transferred to the civil department. By order of Government all surgeons newly joining the establishment are posted to the military department, and under the present regulations have to remain there for two years before their services can be made available for civil employ. Although their number is annually computed according to the requirements of both the military and civil departments, yet there is often the greatest difficulty in obtaining medical officers for civil duty. The result has been that assistant-surgeons have had to be placed in medical charge of some civil stations, and even districts, involving duties of administration and supervision, which they are not qualified to perform. The military operations in Burma are largely responsible for this condition of things, not that the surgeon-general's contention that, making every allowance for these, it must still be admitted that "some ought to be available for civil duty" is indisputable. The matter is receiving the attention of Government, and it is to be hoped some solution of the question may soon be found.

With reference to the payment of midwives, partly by pay and partly by results, it is urged that the results' system should be adopted by every district board and municipality. In the majority of cases they at present receive, in addition to their monthly pay, a remuneration varying from four annas to one rupee for every case in excess of a fixed minimum; in some districts consolidated pay is given. The surgeon-general's recommendation for the adoption of the payment by results' system, as an incentive to good work, is to be communicated to the Local and Municipal Department, by whom we hope it will be adopted.

The financial statements do not call for any special comment.

